# Physical Activity Questionnaires for Pregnancy: A Systematic Review of Measurement Properties

**DOI:** 10.1007/s40279-018-0961-x

**Published:** 2018-08-09

**Authors:** Matteo C. Sattler, Johannes Jaunig, Estelle D. Watson, Mireille N. M. van Poppel, Lidwine B. Mokkink, Caroline B. Terwee, Pavel Dietz

**Affiliations:** 1 Institute of Sport Science, University of Graz, Graz, Austria; 2 Centre for Exercise Science and Sports Medicine, Faculty of Health Sciences, School of Therapeutic Sciences, University of Witwatersrand, Private Bag 3, Johannesburg, 2050 South Africa; 3 MRC/Wits Developmental Pathways for Health Research Unit, Department of Paediatrics, Faculty of Health Sciences, School of Clinical Medicine, University of Witwatersrand, Private Bag 3, Johannesburg, 2050 South Africa; 4 Department of Public and Occupational Health, Amsterdam Public Health Research Institute, VU University Medical Center, Amsterdam, The Netherlands; 5 Department of Epidemiology and Biostatistics, VU University Medical Center, Amsterdam, The Netherlands; 6 Institute of Occupational, Social and Environmental Medicine, University Medical Centre, University of Mainz, Mainz, Germany

## Abstract

**Background:**

In order to assess physical activity (PA) during pregnancy, it is important to choose the instrument with the best measurement properties.

**Objectives:**

To systematically summarize, appraise, and compare the measurement properties of all self-administered questionnaires assessing PA in pregnancy.

**Methods:**

We searched PubMed, Embase, and SPORTDiscus with the following inclusion criteria: (i) the study reported at least one measurement property (reliability, criterion validity, construct validity, responsiveness) of a self-administered questionnaire; (ii) the questionnaire intended to measure PA; (iii) the questionnaire was evaluated in healthy pregnant women; and (iv) the study was published in English. We evaluated results, quality of individual studies, and quality of evidence using a standardized checklist (Quality Assessment of Physical Activity Questionnaires [QAPAQ]) and the GRADE (Grading of Recommendation, Assessment, Development, and Evaluation) approach.

**Results:**

Seventeen articles, reporting 18 studies of 11 different PA questionnaires (17 versions), were included. Most questionnaire versions showed insufficient measurement properties. Only the French and Turkish versions of the Pregnancy Physical Activity Questionnaire (PPAQ) showed both sufficient reliability and construct validity. However, all versions of the PPAQ pooled together showed insufficient construct validity. The quality of individual studies was usually high for reliability but varied considerably for construct validity. Overall, the quality of evidence was very low to moderate.

**Conclusions:**

We recommend the PPAQ to assess PA in pregnancy, although the pooled results revealed insufficient construct validity. The lack of appropriate standards in data collection and processing criteria for objective devices in measuring PA during pregnancy attenuates the quality of evidence. Therefore, research on the validity of comparison instruments in pregnancy followed by consensus on validation reference criteria and standards of PA measurement is needed.

**Electronic supplementary material:**

The online version of this article (10.1007/s40279-018-0961-x) contains supplementary material, which is available to authorized users.

## Key Points

**Table:** 

There was high-quality evidence that the Pregnancy Physical Activity Questionnaire (PPAQ) has sufficient reliability in assessing total physical activity (PA) and vigorous PA (VPA) in pregnancy. However, the questionnaire revealed insufficient construct validity in assessing these scores, but the evidence for this was of low-to-moderate quality.
The Australian Women’s Activity Study (AWAS), Leisure-Time Exercise Questionnaire (LTEQ), Leisure-Time Physical Activity Questionnaire (LTPAQ), and Recent Physical Activity Questionnaire (RPAQ) showed both insufficient reliability and construct validity when assessing either total PA, moderate-to-vigorous PA, or VPA in pregnancy. This assessment was based on very low-to-moderate quality evidence.
Most importantly, we need more high-quality evidence regarding the validity of objective measures of PA in pregnancy, such as accelerometers, and standards in data collection and processing criteria of these devices. Only then will we be able to guarantee adequate and comparable estimations of the validity of a PA questionnaire in pregnancy.

## Introduction

Physical activity (PA) plays a pivotal role in the improvement and maintenance of physical and mental health [1]. In pregnancy, regular PA can have various health benefits for mother and fetus, such as reduced symptoms of depression [2] and lower risks for excessive gestational weight gain [3], gestational diabetes mellitus [4], lower birth weight [5], preterm birth [3], and pre-eclampsia [6]. There is even evidence that PA during pregnancy may improve cardiac and neurobehavioral maturation of the offspring [7], which is in harmony with the premise of fetal programming [8]. Therefore, the American College of Obstetricians and Gynecologists [9] recommends that pregnant women, in the absence of medical or obstetric complications, participate in moderate-intensity activities for at least 20–30 min per day on most or all days of the week.

Research on PA in pregnancy has grown steadily over the last years. To provide solid evidence-based recommendations, and to determine the health benefits of PA, effectiveness of PA interventions, dose-response relationships of PA, and health outcomes, as well as to assess global trends of PA over time, adequate measurement of PA in pregnancy is essential. In particular, a measurement instrument should provide reliable and valid estimates of PA in this target population.

Questionnaires are a commonly used, inexpensive, and acceptable method to determine PA levels. Because of different study purposes, populations, settings, or unsatisfactory pre-existing questionnaires, many PA questionnaires have been developed, which introduces complexity when choosing the right questionnaire for one’s study purpose. Moreover, using different questionnaires hinders the comparability of PA levels across studies and countries, especially if the questionnaires vary in their measurement quality. Therefore, an overview of measurement properties of PA questionnaires for use in pregnancy is helpful to select the best qualified questionnaire. A critical appraisal of the methodological quality of these validation studies and the overall evidence is essential for drawing unbiased conclusions about measurement properties.

Although the measurement properties of PA questionnaires have been systematically reviewed for non-pregnant populations [10–12], there is still a lack of knowledge addressing this issue in pregnancy. The purpose of this systematic review was to critically appraise, compare, and summarize the measurement properties (reliability, criterion validity, construct validity, responsiveness) of all available self-administered questionnaires measuring PA in pregnancy, taking the methodological quality of these studies as well as the quality of evidence into account.

## Methods

### Literature Search

We performed a systematic literature search using a priori defined eligibility criteria in the databases PubMed, Embase using the filter Embase only, and SPORTDiscus. The search strategy included (variations of) the terms ‘physical activity’, ‘measurement properties’ [13], ‘questionnaire’ and ‘pregnancy’ (see Electronic Supplementary Material Appendix S1 for the full search strategy). Publication types such as interviews, case reports, or biographies were excluded. This search strategy was adapted for Embase and SPORTDiscus following their individual search guidelines. Additional studies were identified by searching references of the retrieved articles. The search was performed on the 17 July 2017.

### Eligibility Criteria

The eligibility criteria were based on the previous series of reviews on PA questionnaires [10–12], and adapted to our target population. The following inclusion criteria were used:

(i) The aim of the study was to evaluate one or more of the following measurement properties of a self-administered questionnaire: reliability, criterion validity, construct validity, or responsiveness.
(ii) The aim of the questionnaire was to measure PA, which was defined as any bodily movement produced by skeletal muscles that resulted in energy expenditure (EE) above resting level [14].
(iii) The study was performed in healthy pregnant women, irrespective of the population for which the questionnaire was originally developed (e.g., pregnant women, general population, adolescents).
(iv) The article had to be published in English.

Since different modes of data collection likely cause heterogeneity in effect estimates and data quality [15], the aim of this review was to provide evidence-based recommendations only for self-administered PA questionnaires. Consequently, we excluded PA interviews (face-to-face, telephone), diaries, interview-administered questionnaires, questionnaires measuring physical functioning, and questionnaires (questions) asking about sweating. All studies performed in patients (e.g., pregnant women with gestational diabetes) were excluded. There were no limitations concerning the mean age or body mass index of the study populations.

Finally, measurement properties regarding the internal structure (structural validity, internal consistency, cross-cultural validity/measurement invariance), development, and content validity of the PA questionnaires were not assessed in this review. The evaluation of internal structure (e.g., using Cronbach’s alpha) is relevant for constructs consisting of reflective indicators [16]. These indicators are manifestations of the construct and, thus, should be highly correlated with each other. In contrast, PA is represented by causal or composite indicators, which can independently contribute to PA. The evaluation of content validity would require the inclusion of studies of the development and translations of the questionnaire as well as studies focusing on content validity and expert opinions. Therefore, a single but comprehensive evaluation of content validity of (all available) PA questionnaires should be performed in a future review.

### Selection of Articles

Two researchers independently performed abstract selection, selection of full-text articles, data extraction, and quality assessment. Disagreements were discussed and resolved. Full-text articles were retrieved if the abstracts fulfilled the inclusion criteria or if the abstract did not contain measurement properties, but these were likely to be presented in the full-text article.

### Data Extraction

We used a standardized extraction form, based on the QAPAQ (Quality Assessment of Physical Activity Questionnaire) checklist [17], to obtain the required information to evaluate the methodological quality and results of each individual study. The QAPAQ checklist was developed for PA questionnaires and is based on the COSMIN (COnsensus based Standards for the selection of health Measurement INstruments) checklist for assessing the methodological quality of studies of measurement properties of patient-reported outcome measures (PROMs) [18] and a list of criteria for sufficient measurement properties [19].

To provide a description of the PA questionnaire, the following information was collected: (i) target population of the questionnaire; (ii) dimension(s) of PA (e.g., habitual, EE); (iii) setting (e.g., household, sports); (iv) recall period; (v) number of questions; (vi) parameters of PA (e.g., frequency, duration, intensity); (vii) number and type of scores which can be calculated (e.g., total EE, minutes of activity per day). To assess the methodological quality and results of each individual study, we extracted information regarding study population, sample size, time intervals, data analysis, and results of the measurement properties.

### Assessment of Measurement Properties

#### Content Validity

Content validity is the degree to which the questionnaire encompasses all relevant aspects and dimensions of the intended construct. Since there is no statistical criterion (e.g., numerical value) for content validity, we evaluated content validity for all included questionnaires using the extracted qualitative attributes. Based on previous systematic reviews [11], the following two criteria were assessed: (i) if the questionnaire aims to measure total PA, it should incorporate activities in all settings (home, recreation, sports, transport, work); (ii) the questionnaire should measure at least frequency and duration of PA together with a recall period of at least 1 week.

#### Reliability

Reliability is the extent to which the scores for participants, who did not change, are the same for repeated measurements under several conditions (free from measurement error) [20]. We considered parameters of reliability (Pearson/Spearman correlation, intraclass correlation coefficient [ICC], kappa, concordance) and measurement error (standard error of measurement [SEM], change in the mean or mean difference [$\bar{d}$; systematic error], limits of agreement [LOA; random error], smallest detectable change [SDC], coefficient of variation [CV]) for the assessment of reliability [17].

To ensure that a measurement detects clinically important changes accurately (beyond measurement error), a definition of minimal important change (MIC) of PA is required. Currently, there is no consensus about MIC of PA in pregnancy but a change in the frequency of twice per week or a change in moderate PA or moderate-to-vigorous PA (MVPA) of 30 min (≥ 90 MET [metabolic equivalent of tasks] min) per week can be seen as important for both the individual and the clinician. According to this definition, the PA questionnaire should be able to reliably measure changes of ± 20% of currently recommended PA guidelines (i.e., 150 min of MVPA). Only when the LOA or SDC are smaller than the MIC can one be confident that changes as large as the MIC reflect true changes (e.g., statistically significant) in individual people that cannot be attributed to measurement error. Consequently, measurement error was rated using MIC_frequency_ = 2 and MIC_duration/intensity_ = 30 min (90 MET min) per week. It is important to note that these considerations about MIC were made irrespective of individual differences such as fitness, physical capacity, and body composition. Furthermore, for a CV *(*i.e, standard deviation in relation to the mean), a maximum value of 15% was considered acceptable, which indicates that every observed PA score could vary on average ± 15% of the mean score (or 95% of the observed PA scores were between ± 1.96 × 15% of the mean). Finally, we considered ICC, kappa, and concordance coefficients of ≥ 0.70 or Pearson/Spearman correlation coefficients of ≥ 0.80 as sufficient [17].

Based on QAPAQ [17], each result received either a positive (sufficient), negative (insufficient), or indeterminate rating. The result was sufficient (+) if ICC/kappa/concordance was ≥ 0.70 or Pearson/Spearman ≥ 0.80 or MIC > LOA/SDC or CV ≤ 15%, and otherwise insufficient (–). If no such coefficient was reported, the rating of the result was indeterminate (?).

#### Construct and Criterion Validity

Construct validity is the degree of agreement between the questionnaire and comparable measures of PA, whereas criterion validity is the degree of agreement between the questionnaire and the gold standard of measuring PA. Although doubly-labeled water (DLW) and the respiratory chamber can be considered as the gold standard for measuring EE, there is no gold standard for the assessment of PA. Consequently, all comparisons to other instruments were considered as evidence for construct validity in our review.

Based on QAPAQ [17] and the series of previous systematic reviews [10–12], a priori defined correlations were considered as sufficient (Table 1). The result was sufficient (+) if the correlation was equal to or above the defined cut points, and otherwise insufficient (–). If no correlation coefficient or comparable measure was reported, the rating of the result was indeterminate (?).

**Table 1:** Cut points for sufficient correlations per dimension of PA measured by the questionnaire and level of quality

Dimension	Level 1	Level 2	Level 3
Total PAEE [METs]	Doubly labelled water ≥ 0.70	Accelerometer total counts or average counts ≥ 0.50	Diary, logbook, other questionnaire, interview ≥ 0.70; pedometer steps ≥ 0.40; accelerometer time in moderate, moderate-to-vigorous or vigorous intensity ≥ 0.40
Total PA [min; score]	Accelerometer total counts or average counts ≥ 0.50	Accelerometer time in moderate-to-vigorous intensity ≥ 0.40	Diary, logbook, other questionnaire, interview ≥ 0.70; pedometer steps ≥ 0.40
By intensity			
Vigorous	Accelerometer time in vigorous intensity ≥ 0.50	Accelerometer total counts or average counts ≥ 0.40	Diary, logbook, other questionnaire, interview ≥ 0.70; accelerometer time in light, moderate or moderate-to-vigorous intensity ≥ 0.40; pedometer steps ≥ 0.40
Moderate-to-vigorous	Accelerometer time in moderate-to-vigorous intensity ≥ 0.50	Accelerometer total counts or average counts ≥ 0.40	Diary, logbook, other questionnaire, interview ≥ 0.70; accelerometer time in light, moderate or vigorous intensity ≥ 0.40; pedometer steps ≥ 0.40
Moderate	Accelerometer time in moderate intensity ≥ 0.50	Accelerometer total counts or average counts ≥ 0.40	Diary, logbook, other questionnaire, interview ≥ 0.70; accelerometer time in light, moderate-to-vigorous or vigorous intensity ≥ 0.40; pedometer steps ≥ 0.40
Light	Accelerometer time in light intensity ≥ 0.50	Accelerometer total counts or average counts ≥ 0.40	Diary, logbook, other questionnaire, interview ≥ 0.70; accelerometer time in moderate, moderate-to-vigorous or vigorous intensity ≥ 0.40; pedometer steps ≥ 0.40
By type			
Walking	Pedometer or accelerometer walking total counts ≥ 0.70	–	Diary, logbook, other questionnaire, interview ≥ 0.70
Leisure time	Accelerometer total counts in leisure time ≥ 0.50	Accelerometer total counts or average counts ≥ 0.40	Diary, logbook, other questionnaire, interview ≥ 0.70; accelerometer time in moderate, moderate-to-vigorous or vigorous intensity ≥ 0.40; pedometer steps ≥ 0.40
Occupational	Direct observational method ≥ 0.60 Accelerometer total counts during working hours ≥ 0.50	Accelerometer total counts or average counts ≥ 0.40	Diary, logbook, other questionnaire, interview ≥ 0.70; accelerometer time in light, moderate, moderate-to-vigorous or vigorous intensity ≥ 0.40; pedometer steps ≥ 0.40
Household/caregiving	Accelerometer time in light, light-to-moderate or moderate intensity ≥ 0.50	Accelerometer total counts or average counts ≥ 0.40	Diary, logbook, other questionnaire, interview ≥ 0.70; accelerometer time in moderate-to-vigorous or vigorous intensity ≥ 0.40; pedometer steps ≥ 0.40
Sports/exercise	Accelerometer time in moderate-to-vigorous or vigorous intensity ≥ 0.50	Accelerometer total counts or average counts ≥ 0.40	Diary, logbook, other questionnaire, interview ≥ 0.70; accelerometer time in light or moderate intensity ≥ 0.40; pedometer steps ≥ 0.40

*METs* metabolic equivalent of tasks, *min* minutes, *PA* physical activity, *PAEE* physical activity energy expenditure

#### Responsiveness

Responsiveness can be considered as an aspect of validity and is the degree to which an instrument detects changes over time in the construct [21, 22]. In this case, it is the ability of the questionnaire to detect changes in PA in a longitudinal setting (validity of change score rather than single score). We applied the same approach as for construct validity to rate responsiveness, except that the change in scores of the questionnaire was compared with the change in scores of other instruments such as accelerometers.

### Quality of Individual Studies

Evaluation of the methodological quality of the included studies was based on the QAPAQ checklist [17], the series of previous reviews [10–12], as well as the recently updated COSMIN checklist [23]. For the assessment of the quality of all individual studies, we assigned one of three different levels of quality (1: very good, 2: adequate, 3: doubtful) for each outcome (PA score) and measurement property. If an individual study had any substantial flaws in the design or analysis, the quality was inadequate (level 4).

To evaluate the methodological quality of studies of reliability and measurement error, we considered ICC, kappa, and concordance as adequate measures of reliability, and LOA, SDC, and CV as adequate measures of measurement error. We considered Pearson and Spearman correlation coefficients as less adequate since they neglect systematic errors between measurements [24]. However, Pearson and Spearman correlations are widely used in validation studies and, thus, were not omitted from our review. To ensure that the measured construct did not change over time, an adequate time interval between test and retest should be defined. For pregnancy, we considered a time interval from 2 days to 2 weeks as adequate to ensure that PA did not change over time (e.g., between the second and third trimesters) [2]. If there have been no substantial flaws in the design or analysis (level 4), we assigned one of the following levels of quality for each PA score reported in an individual study for the assessment of reliability and measurement error:

- Level 1: an adequate time interval between test and retest (2 days–2 weeks) and reporting of ICC, LOA, SDC, SEM, CV, kappa, or concordance.
- Level 2: an inadequate time interval between test and retest (> 2 weeks) and reporting of ICC, LOA, SDC, SEM, CV, kappa, or concordance; or an adequate time interval between test and retest (2 days–2 weeks) and reporting of Pearson/Spearman correlation.
- Level 3: an inadequate time interval between test and retest (> 2 weeks) and reporting of Pearson/Spearman correlation.

To evaluate the methodological quality of studies of construct validity and responsiveness, it is important to formulate a priori hypotheses about the expected direction and magnitude of the results, which guarantees unbiased conclusions. Since this criterion was rarely met previously [10–12] and a study may still provide unbiased coefficients without these hypotheses, we did not rate the quality of these studies as inadequate but stated how many studies formulated such an a priori hypothesis. We further applied our own criteria in order to compare all results with the same set of hypotheses. Depending on the type of comparison, we assigned three different levels of quality for the assessment of construct validity and responsiveness (Table 1). Higher levels of quality (level 1 or 2) were provided if the questionnaire was evaluated against objective measures of PA (e.g., accelerometer) depending on the use of the objective data. More specifically, a higher level of quality was given the more similar the constructs were. For example, the comparison of moderate PA from the questionnaire with moderate PA from the accelerometer is currently the optimal approach (level 1), whereas a comparison with total counts (including, light, moderate, and vigorous PA [VPA]) is less optimal (level 2). We assigned level 3 of quality when the questionnaire was compared with measures less similar to the construct, such as pedometers, questionnaires, diaries, and interviews, or if different intensity levels were compared against each other (e.g., light PA estimated from the questionnaire compared with MVPA estimated from the accelerometer).

### Quality of Evidence

We evaluated the quality of the body of evidence using the state of-the-art GRADE (Grading of Recommendation, Assessment, Development, and Evaluation) approach [25]. Since this assessment should be outcome-specific, we evaluated the quality of evidence for each questionnaire version (including different language versions) and measurement property (reliability, measurement error, construct validity, responsiveness) for three outcomes (total PA, MVPA, and VPA) separately. In addition, we pooled the evidence from individual studies when there was more than one study of the same questionnaire available. In particular, we applied a modified GRADE approach to grade the body of evidence [26]. For each outcome (PA score), the quality of evidence could be high, moderate, low, or very low depending on the assessment of four factors (risk of bias [methodological quality of the individual study], imprecision, inconsistency, indirectness). At the beginning, the quality of evidence for each outcome was high, but could be downgraded if there were any serious shortcomings in these factors. Currently, there are no guidelines for upgrading due to very good measurement properties.

Regarding *risk of bias*, high-quality evidence (no downgrading) was available when most individual studies had very good quality (level 1). When most individual studies were of doubtful quality (level 3) or only one study of adequate (level 2) or very good quality was available, we downgraded the quality of evidence by one level (e.g., from moderate to low). When only one individual study of doubtful quality or multiple studies of inadequate quality (level 4) were available, we downgraded by two levels. Moreover, we downgraded by three levels if there was only one individual study of inadequate quality available. To evaluate *imprecision*, we determined the optimal information size (OIS) to ensure a sufficient precision in the estimation of adequate effect sizes. Assuming that ICC = 0.7, a sample size of *n* ≥ 45 would be required to obtain a 95% confidence interval (CI) with a maximum width of 0.30 (i.e., ± 0.15; calculated using STATA 12.1, Statacorp, College Station, TX, USA) [27]. Likewise, assuming *r* = 0.40, a sample size of *n* ≥ 123 would be required to obtain a 95% CI with the same width [28]. Serious imprecision was present if the total sample size did not meet these criteria (i.e., 45 for reliability and 123 for construct validity and responsiveness), and we downgraded the quality of evidence by one level. We downgraded the quality of evidence by two levels (very serious imprecision) when the total sample size was *n* < 12 for reliability or *n* < 32 for construct validity and responsiveness (95% CI width of ± 0.30). Because publication bias is difficult to assess in studies of measurement properties (e.g., lack of registries), we did not downgrade due to this factor. Finally, we downgraded by one or two levels in the presence of unexplained *inconsistency* (differences in results [i.e., sufficient, insufficient]) or *indirectness* (differences in populations, interventions, outcomes, indirect comparisons).

## Results

### Literature Search

**Fig. 1:**
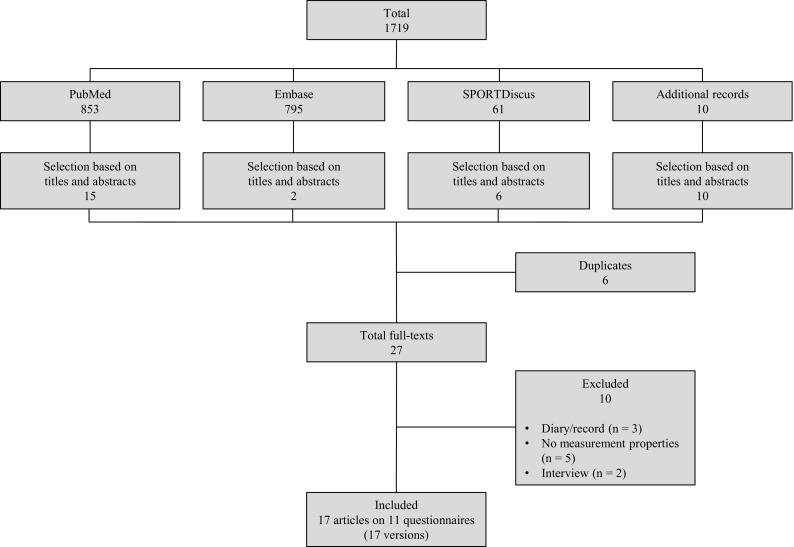
Flowchart of literature search and study selection

The literature search resulted in 1,719 hits. Of these, 27 articles were selected based on titles and abstracts. After reading the full-texts, ten articles were excluded because of the absence of measurement properties (*n* = 5) [29–33] or using a diary/record (*n* = 3) [34–36] or an interview (*n* = 2) [37, 38]. Finally, 17 articles [39–55] on 11 different PA questionnaires (17 versions) [39, 44, 56–63] were included (Fig. 1). Overall, these 17 articles reported 18 studies of measurement properties. It should be noted that the studies describing the development of the short and long form of the International Physical Activity Questionnaire (IPAQ) [59] share the same reference in order to avoid any misconceptions. All results are presented for questionnaires developed for the pregnant and non-pregnant population separately only to improve readability.

Table 2 shows a summary of all included articles and questionnaires in combination with evaluated measurement properties and study populations. Construct validity was assessed for all questionnaires, whereas reliability (parameters of reliability and measurement error) was assessed for six questionnaires (11 versions) and responsiveness for two questionnaires. In most studies, an accelerometer was used as a comparison measure. Eight studies [42–46, 49, 51, 55] assessed the measurement properties of the Pregnancy Physical Activity Questionnaire (PPAQ) [44] or adaptations of this questionnaire (e.g., Japanese version). Another study [48] evaluated the long form of the IPAQ, whereas two studies (of reliability and construct validity), reported in one article [52], evaluated the short form of the IPAQ (IPAQ-SF). One study [39] used a strongly modified version of the IPAQ measuring leisure time (LT) PA (LTPA) in pregnancy. One article [40] reported one study evaluating two questionnaires, namely the Australian Women’s Activity Study (AWAS) [60] and the Recent Physical Activity Questionnaire (RPAQ) [57].

**Table 2:** Explanation of acronyms or abbreviated names of questionnaires, studies on measurement properties and sample characteristics

Abbreviation	Full name of questionnaire	Studies on measurement properties	Assessed measurement properties			Comparison measures	Sample *N* (of consented), age (years), gestational week, pre-pregnancy BMI (kg/m^2^), specific characteristics, nationality
			Reliability	Validity	Responsiveness		
AQuAA	Activity Questionnaire for Adolescents and Adults [58]	Oostdam et al. [50]		●	●	Acc	55 (of 124), mean age = 31.4 (SD = 3.9), gw = 17, 24 and 32, mean BMI = 33.7 (SD = 5.3), overweight and obese, Netherlands
AWAS	Australian Women’s Activity Study [60]	Bell et al. [40]	●	●		Acc	59 (N/A), median age = 31.0 (IQR = 26.0–34.0), median gw = 12, median BMI^a^ = 28.6 (IQR = 26.6–33.0), overweight and obese, UK
GPAQ	Global Physical Activity Questionnaire [56]	Watson et al. [54]		●	●	Acc	95 (of 150), mean age = 29.5 (SD = 5.7), mean BMI^a^ = 26.9 (SD = 5.0), all trimesters, South Africa
IPAQ	International Physical Activity Questionnaire (long-form) [59]	Harrison et al. [48]		●		Acc, Ped	30 (of 48), mean age = 33.6 (SD = 4.7), gw = 26–28, mean BMI^a^ = 31.2 (SD = 5.1), Australia
IPAQ-SF	International Physical Activity Questionnaire (short-form) [59]	Sanda et al. [52]	●	●		Acc	Reliability study: 88 (of 154), median age = 28 (range = 20–42), median gw = 19 (range = 16–31), median BMI = 22.6 (range = 17.9–38.3), Norway Validity study: 64 (of 118), median age = 30 (range = 22–44), median gw = 21 (range = 16–35), median BMI = 22.3 (range = 19.5–43.2), Norway
LTEQ	Leisure-Time Exercise Questionnaire [61]	Symons Downs et al. [53]	●	●		Ped	30 (of 37), mean age = 31.0 (SD = 4.1), gw = 20 and 32, mean BMI^a^ = 25.1/26.5 (SD = 5.2/5.4), Study 2, USA
LTPAQ	Leisure-Time Physical Activity Questionnaire (modified from IPAQ) [39]	Aittasalo et al. [39]	●	●		Ped, Log	49 (of 79); mean age = 29.2 (SD = 5.5); mean gw = 23.5 (SD = 6.4), mean BMI = 25.8 (SD = 5.3), Finland
PAPQ	Physical Activity and Pregnancy Questionnaire [62]	Haakstad et al. [47]		●		Acc	77 (of 82), mean age = 32.3 (SD = 3.6), mean gw = 34.7 (SD = 2.1), mean BMI = 22.3 (SD = 2.2), Norway
PPAQ	Pregnancy Physical Activity Questionnaire [44]	Cohen et al. [46] Bilingual version (English, French)		●		Ped	61 (of 81), mean age = 30.0–34.5, gw (second/third) = 21.1/32.3, BMI = 70% normal pre-pregnancy BMI, Canada
		Xiang et al. [55] Chinese version	●	●		Acc	182 (of 224), mean age = 27.5 (SD = 4.1), gw = 22.4 (SD = 8.6), mean BMI = 21.1 (SD = 2.9), all trimesters, China
		Brett et al. [42] English version		●		Acc	29 (N/A), mean age (active/non-active) = 31.0 (SD = 3)/32.0 (SD = 3), mean gw (active/non-active) = 24.8 (SD = 2.6)/25.4 (SD = 1.9), mean BMI (active/non-active) = 23.0 (SD = 3.2)/25.0 (SD = 5.8), Canada
		Chasan-Taber et al. [44] English version	●	●		Acc	54 (of 63), age range (inclusion) = 16.0–40.0, all trimesters, USA
		Chandonnet et al. [43] French version	●	●		Acc	49 (of 56), mean age = 29.8 (SD = 4.2), mean gw (all trimesters) = 24.7 (SD = 9.0), mean BMI = 34.7 (SD = 5.1), obese, Canada
		Matsuzaki et al. [49] Japanese version	●	●		Acc	58 (of 60), mean age = 32.9 (SD = 3.9), mean BMI = 20.6 (SD = 3.2), all trimesters, Japan
		Cirak et al. [45] Turkish version	●	●		Q, Ped	204 (of 204), mean age = 28.2 (SD = 4.9), mean gw = 22.5 (SD = 11.0), mean BMI = 23.3 (SD = 4.1), all trimesters, Turkey
		Ota et al. [51] Vietnamese version	●	●		Ped	60 (of 60), mean age = 26.8 (SD = 5.0), mean BMI^a^ = 21.3 (SD = 2.5), all trimesters, Vietnam
Q1 of MoBa	Questionnaire of recreational exercise from Norwegian Mother and Child Cohort Study [63]	Brantsæter et al. [41]		●		Acc	112 (of 119), mean age = 31.2 (SD = 4.0), mean gw = 20, mean BMI^a^ = 24.8 (SD = 3.5), Norway
RPAQ	Recent Physical Activity Questionnaire [57]	Bell et al. [40]	●	●		Acc	59 (N/A), median age = 31.0 (IQR = 26.0–34.0), median gw = 12, median BMI^a^ = 28.6 (IQR = 26.6–33.0), overweight and obese, UK

*Acc* accelerometer, *BMI* body mass index, *gw* gestational week, *IQR* interquartile range, *Log* Logbook, *N/A* not applicable, *Ped* pedometer, *Q* questionnaire, *SD* standard deviation, *UK* United Kingdom, *USA* United States of America

^a^Pregnancy BMI (e.g., at point of recruitment or time of measuring)

### Description of Questionnaires

A detailed description of the questionnaires is shown in Table 3. Of the 11 questionnaires, four were developed to assess PA in pregnant women [39, 44, 62, 63], whereas five were developed for adults [56, 57, 59, 61], one for adults and adolescents [58], and one for women with young children [60].

**Table 3:** Description of PA questionnaires

Questionnaire	Target population	Construct				Format		
		Dimension	Setting	Recall period	No. of questions	Parameters	Scores	Unit of measurement
AQuAA [58]	Adolescents/young Adults	PA, SB	Commuting, work/school, household, leisure, sports	Past week	19	F, D, I	Total, light, moderate, vigorous, SB	MET-min/wk
AWAS [60]	Women with young children	PA	Planned activities, employment, childcare, domestic responsibilities, transportation	Typical week (in the past month)	68	F, D, I	Total, light, moderate, vigorous, SB, brisk walking	Days/wk, min/day
GPAQ [56]	General population	PA	Recreation/sports/leisure, occupational (paid and unpaid), transportation, SB	Typical week	16	F, D, I	Total, moderate, vigorous, SB, recreation/sports/leisure, occupational, transportation	MET-min/wk
IPAQ [59]	Adults	PA	Recreation/sports/leisure, household, yard/garden, occupational, transportation, SB	Last 7 days/usual week	31 (27 in latest version[87])	F, D, I	Total, light/walking, moderate, vigorous, SB, leisure, household, occupational, transportation	MET-min/wk
IPAQ-SF [59]	Adults	PA	All settings, SB	Last 7 days/usual week	7	F, D, I	Total, moderate, vigorous, SB, walking	MET-min/wk
LTEQ [61]	Adults	LT Exercise	Leisure exercise	Typical 7-day period (week)	4	F, I	Total, mild, moderate, strenuous	Frequency/wk
LTPAQ [39]	Pregnant women	LTPA	Leisure, household	Average of previous 2 weeks	N/A	F, D, I	Total LTPA^a^, light LTPA^a^, moderate LTPA^a^, vigorous LTPA^a^	Min/wk, sessions/wk
PAPQ [62]	Pregnant women	PA	Recreation/sports/leisure, occupational, transportation, household/caregiving, SB	Trimester-specific	53	F, D, I	Total, standing/moving, light, moderate, high, SB	Min/wk, min/day
PPAQ [44]	Pregnant women	PA	Household/caregiving, occupational, sports/exercise, transportation, inactivity	Current trimester	32	F, D, I	Total, light, moderate, vigorous, SB, household/caregiving, occupational, sports/exercise	MET-h/wk
Q1 of MoBa [63]	Pregnant women	Recreational exercise	Recreational exercise	Since becoming pregnant	14	F	Total	Frequency/wk, MET-min/wk^b^
RPAQ [57]	Adults	PA	Stair climbing at home, occupational, transportation, leisure, SB	Last 4 weeks	51	F, D, I	Total, light, moderate, vigorous, SB, home, occupational, transportation, leisure	MET-h/day

*AQuAA* Activity Questionnaire for Adolescents and Adults, *AWAS* Australian Women’s Activity Study, *D* duration, *F* frequency, *GPAQ* Global Physical Activity Questionnaire, *h* hours, *I* intensity, *IPAQ* International Physical Activity Questionnaire (long-form), *IPAQ-SF* International Physical Activity Questionnaire (short-form), *min* minutes, *LT* leisure time, *LTEQ* Leisure-Time Exercise Questionnaire, *LTPAQ* Leisure Time Physical Activity Questionnaire (modified from IPAQ), *MET* metabolic equivalent of task, *N/A* not applicable, *PA* physical activity, *PAPQ* Physical Activity and Pregnancy Questionnaire, *PPAQ* Pregnancy Physical Activity Questionnaire, *Q1 of MoBa* Questionnaire of recreational exercise from Norwegian Mother and Child Cohort Study, *RPAQ* Recent Physical Activity Questionnaire, *SB* sedentary behaviour, *wk* week

^a^Scores can be calculated separately for unstructured LTPA (household) and structured LTPA (LT excluding household)

^b^The final version of Q1 of MoBa did not assess duration of PA; however, they imputed information about time spent on each activity from a preliminary version of the questionnaire used in 2555 women to calculate MET-min/wk

Of the seven questionnaires that were developed for the non-pregnant population, six (Activity Questionnaire for Adolescents and Adults [AQuAA], AWAS, Global Physical Activity Questionnaire [GPAQ], IPAQ, IPAQ-SF, RPAQ) aim to measure the construct PA and one (Leisure-Time Exercise Questionnaire [LTEQ]) measures LT exercise. When assessing (total) PA, the AQuAA, AWAS, GPAQ, IPAQ, and RPAQ cover all relevant settings of PA (home, recreation, sports, transport, work). The GPAQ assesses sport-related PA within discretionary time (leisure, recreation, sports). Likewise, the RPAQ assesses sport-related PA such as competitive running and swimming in its section on recreation. The AWAS assesses planned activities (including sports, leisure, recreation) and was developed to measure PA in women with young children, and therefore focuses particularly on childcare activities and domestic responsibilities. The IPAQ-SF aims to cover all settings of PA without discriminating between them. Most of the questionnaires use a typical week or the last week as a recall period and the number of questions varies from seven (IPAQ-SF) to 68 (AWAS). Duration, frequency, and intensity of PA are obtained by all questionnaires except LTEQ, which only collects frequency and intensity. Usually, both a total PA score and separate scores for time spent in different intensity levels (e.g., light PA, VPA) as well as sedentary behavior (SB) can be calculated using minutes per day/week, MET min per week or frequency per week as units of measurement. In addition, GPAQ, IPAQ, and RPAQ provide separate PA scores for different settings.

Of the four questionnaires developed for the pregnant population, PA is measured with reference to the specific trimester (Physical Activity and Pregnancy Questionnaire [PAPQ], PPAQ), the last 2 weeks (Leisure-Time Physical Activity Questionnaire [LTPAQ]) [39] or since becoming pregnant (Questionnaire of recreational exercise from Norwegian Mother and Child Cohort Study [Q1 of MoBa]) [63]. PAPQ and PPAQ aim to measure the construct (total) PA, whereas LTPAQ and Q1 of MoBa aim to measure LTPA or recreational exercise during pregnancy. The LTPAQ was based on the IPAQ but was strongly modified to provide a better discrimination between the structured (LT excluding household) and unstructured (household) features of PA. Parameters of duration, frequency, and intensity of PA are assessed by all questionnaires except Q1 of MoBa. Scores for total PA, time spent in light PA, moderate PA, VPA, and SB can be calculated for the PAPQ, PPAQ, and LTPAQ. For Q1 of MoBa, only a total PA score can be calculated. All four questionnaires use minutes per week or MET min/week to calculate PA scores.

Finally, all questionnaires that assigned MET intensities for activities use compendium-based information about intensities for different activities [64]. These MET intensities are based on the general population, including men and non-pregnant women. In contrast, the PPAQ uses pregnancy-specific MET intensities whenever possible, such as for walking and light-to-moderate intense household activities [44].

### Assessment of Measurement Properties

#### Content Validity

A comprehensive evaluation of the content validity of PA questionnaires during pregnancy was not part of this review. Consequently, no included study assessed the content validity in a methodological approach but some provided information on content validity. During the development of the PPAQ, one study [44] used 24-h recalls to select both prevalent and discriminatory activities of pregnant women. The findings of the study showed that watching television, standing or slowly walking at work while carrying light/moderate loads, and childcare were the most relevant activities. Another study [54] discussed the content validity of the GPAQ theoretically in the context of previous research and expert opinions. Their conclusion was that the GPAQ includes important settings (e.g., work, transport, leisure) and scores (frequency, duration, intensity) of PA but including pregnancy-specific activities (and settings) such as caregiving might result in a better content validity. Furthermore, one study [39] of the LTPAQ strongly modified the IPAQ to provide a better discrimination between the structured (LT excluding household) and unstructured (household) features of PA. They excluded occupational PA and used the degree of breathlessness (none, some, strong) instead of light, moderate, and vigorous to describe the intensity of activities, which may result in a better understanding for some women. Finally, studies of adaptations of the PPAQ [43, 45, 49, 51, 55] included expert opinions and pilot studies to assess content validity and, consequently, items were modified and/or deleted during their cross-cultural validation process.

According to our criterion (i) (see Sect. 2.5.1), of those questionnaires that aim to measure total PA, AQuAA, AWAS, GPAQ, IPAQ, IPAQ-SF, PAPQ, and PPAQ cover all relevant settings of PA. The RPAQ does not collect information on household-related activities [57] since the authors showed in a previous study [65] that these activities were inversely correlated with objectively measured PA. Therefore, they only included a few activities such as stair-climbing at home, mowing the lawn, watering the lawn or garden, or home maintenance. The IPAQ-SF aims to cover all settings of PA, but domain-specific scores cannot be obtained. The LTEQ, LTPAQ, and Q1 of MoBa were developed to collect specific information about LT/recreational exercise and LTPA rather than total PA. According to criterion (ii) (see Sect. 2.5.1), all included questionnaires assess frequency and duration of PA except LTEQ and Q1 of MoBa and no questionnaire uses a recall period of less than 1 week. In sum, the AQuAA, AWAS, GPAQ, IPAQ, IPAQ-SF, LTPAQ, PAPQ, and PPAQ provided sufficient content validity for the assessment of PA during pregnancy, whereas LTEQ, Q1 of MoBa, and RPAQ did not.

#### Reliability

The results for reliability (parameters of reliability and measurement error) of ten studies of six questionnaires (11 versions) are summarized in Table 4. Of the questionnaires developed for the non-pregnant population, the IPAQ-SF [52] showed sufficient reliability for all estimates of PA, the LTEQ [53] for strenuous LT exercise but not for total, mild, and moderate LT exercise, and the RPAQ [40] showed sufficient reliability for moderate PA but insufficient reliability for all other estimates of PA. The AWAS [40] showed insufficient reliability (ICC < 0.70).

**Table 4:** Parameters of reliability and measurement error of PA questionnaires during pregnancy

Questionnaire	Study population (*n*) for analysis	Interval	Results	Quality and rating^a^
AWAS [40]	56	1 week	Total: *κ* = 0.53 [0.42–0.64]	1−
			Light: *κ* = 0.49 [0.37–0.60]	1−
			Brisk walking: *κ* = 0.51 [0.37–0.64]	1−
			Moderate (excluding brisk walking): *κ* = 0.49 [0.37–0.60]	1−
			Moderate and brisk walking: *κ* = 0.55 [0.44–0.67]	1−
			Vigorous: *κ* = 0.13 [0.00–0.25]	1−
			MVPA: *κ* = 0.57 [0.46–0.68]	1−
			Sedentary: *κ* = 0.42 [0.31–0.53]	
IPAQ-SF [52]	88	2 weeks	Moderate: ICC = 0.81 [0.71–0.88]	1+
			Vigorous: ICC = 0.84 [0.74–0.90]	1+
			MVPA: ICC = 0.81 [0.69–0.89]	1+
LTEQ [53]	37	12 weeks	Total LT exercise: *r* = 0.72	3−
			Mild LT exercise: *r* = 0.69	3−
			Moderate LT exercise: *r* = 0.23	3−
			Strenuous LT exercise: *r* = 0.83	3+
LTPAQ [39]	49	2 weeks	Total LTPA (frequency of sessions): $\bar{d}$= −0.7, LOA^b^ = − 10.7 to 9.3	1−
			LT-MVPA (frequency of sessions): $\bar{d}$= − 0.2, LOA^b^ = − 6.3 to 5.9	1−
			Light LTPA (frequency of sessions): $\bar{d}$= − 0.5, LOA^b^ = − 5.8 to 4.8	1−
			Total LTPA (duration): CV = 119% [92–168]	1−
			LT-MVPA (duration): CV = 225% [167–336]	1−
			Light LTPA (duration): CV = 125% [97–177]	1−
PPAQ [55] Chinese version	125	1 week	Total: ICC = 0.77	1+
			Light: ICC = 0.75	1+
			Moderate: ICC = 0.59	1−
			Vigorous: ICC = 0.28	1−
			Household/caregiving: ICC = 0.74	1+
			Occupational: ICC = 0.75	1+
			Sports/exercise: ICC = 0.34	1−
			Sedentary: ICC = 0.76	
PPAQ [44] English version	54	1 week	Total: ICC = 0.78	1+
			Light: ICC = 0.78	1+
			Moderate: ICC = 0.82	1+
			Vigorous: ICC = 0.81	1+
			Household/caregiving: ICC = 0.86	1+
			Occupational: ICC = 0.93	1+
			Sports/exercise: ICC = 0.83	1+
			Sedentary: ICC = 0.79	
PPAQ [43] French version	49 *n*_occup_ = 20	1 week	Total: ICC = 0.90	1+
			Light: ICC = 0.86	1+
			Moderate: ICC = 0.86	1+
			Vigorous: ICC = 0.81	1+
			Household/caregiving: ICC = 0.89	1+
			Occupational: ICC = 0.84	1+
			Sports/exercise: ICC = 0.82	1+
			Transportation: ICC = 0.59	1−
			Sedentary: ICC = 0.88	
PPAQ [49] Japanese version	58 *n*_occup_ = 24	1 week/2 weeks	Total: ICC_1wk_ = 0.87 [0.79–0.92]; ICC_2wks_ = 0.77 [0.64–0.86]	1+; 1+
			Light: ICC_1wk_ = 0.83 [0.73–0.89]; ICC_2wks_ = 0.76 [0.63–0.85]	1+; 1+
			Moderate: ICC_1wk_ = 0.79 [0.66–0.87]; ICC_2wks_ = 0.71 [0.55–0.82]	1+; 1+
			Household/caregiving: ICC_1wk_ = 0.93 [0.89–0.96]; ICC_2wks_ = 0.84 [0.74–0.90]	1+; 1+
			Occupational: ICC_1wk_ = 0.66 [0.37–0.96]; ICC_2wks_ = 0.84 [0.74–0.90]	1−; 1+
			Sports/exercise: ICC_1wk_ = 0.61 [0.36–0.77]; ICC_2wks_ = 0.56 [0.31–0.74]	1−; 1−
			Transportation: ICC_1wk_ = 0.66 [0.37–0.73]; ICC_2wks_ = 0.58 [0.36–0.76]	1−; 1−
			Inactivity: ICC_1wk_ = 0.74 [0.66–0.87]; ICC_2wks_ = 0.71 [0.55–0.82]	
			Sedentary: ICC_1wk_ = 0.78 [0.66–0.87]; ICC_2wks_ = 0.72 [0.57–0.82)	
PPAQ [45] Turkish version	204	1 week	Total: ICC = 0.95 [0.91–0.97]	1+
			Light: ICC = 0.93 [0.89–0.96]	1+
			Moderate: ICC = 0.96 [0.92–0.98]	1+
			Vigorous: ICC = 0.98 [0.96–0.99]	1+
			Household/caregiving: ICC = 0.96 [0.93–0.98]	1+
			Occupational: ICC = 0.99 [0.99–0.996]	1+
			Sports/exercise: ICC = 0.92 [0.87–0.96]	1+
			Sedentary: ICC = 0.96 [0.93–0.98]	
PPAQ [51] Vietnamese version	60	2 weeks	Total: ICC = 0.88 [0.83–0.94]	1+
			Light: ICC = 0.88 [0.82–0.94]	1+
			Moderate: ICC = 0.90 [0.85–0.95]	1+
			Vigorous: ICC = 0.87 [0.81–0.93]	1+
			Household/caregiving: ICC = 0.92 [0.88–0.96]	1+
			Occupational: ICC = 0.90 [0.85–0.95]	1+
			Sports/exercise: ICC = 0.93 [0.90–0.97]	1+
			Sedentary: ICC = 0.94 [0.90–0.97]	
RPAQ [40]	57	1 week	Total (EE): *κ* = 0.57 [0.46–0.68]	1−
			Total (time): *κ* = 0.67 [0.56–0.79]	1−
			Light: *κ* = 0.65 [0.54–0.76]	1−
			Moderate: *κ* = 0.79 [0.68–0.90]	1+
			Vigorous: *κ* = 0.42 [0.30–0.53]	1−
			MVPA: *κ* = 0.69 [0.58–0.80]	1−
			Sedentary: *κ* = 0.66 [0.55–0.77]	

*AWAS* Australian Women’s Activity Study, *CV* coefficient of variation, $\bar{d}$ change in the mean, *EE* energy expenditure, *ICC* intraclass correlation coefficient, *ICC*_*1wk*_ intraclass correlation coefficient for one week interval, *ICC*_*2wks*_ intraclass correlation coefficient for 2 weeks interval, *IPAQ-SF* International Physical Activity Questionnaire (short-form), *κ* kappa coefficient, *LOA* limits of agreement, *LT* leisure time, *LTEQ* Leisure-Time Exercise Questionnaire, *LTPAQ* Leisure Time Physical Activity Questionnaire (modified from IPAQ), *MVPA* moderate-to-vigorous physical activity, *n*_*occup*_ sample size for occupational physical activity, *PA* physical activity, *PPAQ* Pregnancy Physical Activity Questionnaire, *r* Pearson correlation coefficient, *RPAQ* Recent Physical Activity Questionnaire

^a^As described in Sect. 2.6, the quality of the individual study was evaluated per questionnaire and PA score using four levels (1: very good, 2: adequate, 3: doubtful, 4: inadequate). Additionally, the reported results were rated (i.e., sufficient [+], insufficient [–]) as described in Sect. 2.5.2

^b^${\text{LOA}}\; = \,\bar{d}\, \pm \,1.96 \times s \times \surd 2$, where *s* = within-subject standard deviation (typical error) [88]

Of the questionnaires developed for the pregnant population, parameters of reliability and measurement error were only assessed for (versions of) the PPAQ and LTPAQ. In sum, studies of the English [44], Turkish [45], and Vietnamese versions [51] of the PPAQ showed sufficient reliability. The Chinese version [55] showed sufficient reliability for all PA scores except moderate PA, VPA, and sports/exercise. The French version of the PPAQ [43] showed sufficient reliability for all scores except for transportational PA and, likewise, the Japanese version [49] for all scores except for transportational PA, sports/exercise, and occupational PA (1-week interval only). Although three studies [39, 49, 51] assessed measurement error, only one study reported LOA or CV for repeated measurements. In particular, the results for the LTPAQ [39] were insufficient because of large LOA (MIC_frequency/duration_ < LOA/SDC) and CV. These values indicate large measurement errors and hamper a reliable detection of MIC of PA (e.g., two sessions or 30 min of MVPA per week) [17].

#### Construct and Criterion Validity

The results for construct validity are summarized in Table 5. Of the 11 different questionnaires, construct validity was mostly assessed by validation against accelerometers and less often against pedometers, logbooks, or other PA questionnaires.

**Table 5:** Construct validity and responsiveness of PA questionnaires during pregnancy

Questionnaire	Study population (*n*) for analysis	Comparison measure (type; placement; registration period [valid week]; epoch length; cut points^a^)	Results^b^	Quality and rating^c^
AQuAA [50]	55 *n*_t1_ = 55; *n*_t2_ = 47; *n*_t3_ = 31; *n*_t2–t1_ = 31; *n*_t3–t1_ = 25; *n*_t3–t2_ = 22	Accelerometer (ActiGraph ActiTrainer; waist; waking hours of 4 days [3 days]; 60 s; Freedson et al. [78], Hendelman et al. [79], Swartz et al. [77])	*1st measures*	
			Total: *ρ* = 0.14	1−
			Light: *ρ* = 0.05	1−
			Moderate^d^: *ρ* = 0.05; *ρ* = − 0.02; *ρ* = − 0.03	1−; 1−; 1−
			Vigorous^d^: *ρ* = 0.05; *ρ* = 0.02; *ρ* = 0.22	1−; 1−; 1−
			Sedentary: *ρ* = 0.23	
			*2nd measures*	
			Total: *ρ* = 0.34	1−
			Light: *ρ* = 0.22	1−
			Moderate^d^: *ρ* = − 0.10; *ρ* = − 0.15; *ρ* = − 0.13	1−; 1−; 1−
			Vigorous^d^: *ρ* = − 0.01; *ρ* = − 0.15; *ρ* = − 0.09	1−; 1−; 1−
			Sedentary: *ρ* = 0.19	
			*3rd measures*	
			Total: *ρ* = − 0.10	1−
			Light: *ρ* = − 0.10	1−
			Moderate^d^: *ρ* = 0.06; *ρ* = − 0.03; *ρ* = 0.06	1−; 1−; 1−
			Vigorous^d^: *ρ* = 0.30; *ρ* = 0.19; *ρ* = 0.38	1−; 1−; 1−
			Sedentary: *ρ* = 0.12	
			*Responsiveness*	
			Total: *ρ*_*t*2–t1_ = − 0.03; *ρ*_*t*3–t1_ = − 0.06; *ρ*_*t*3–t2_ = 0.19	1−; 1−; 1−
			Light: *ρ*_*t*2–t1_ = 0.02; *ρ*_*t*3–t1_ = 0.01; *ρ*_*t*3–t2_ = 0.19	1−; 1−; 1−
			Moderate: *ρ*_*t*2–t1_ = − 0.24; *ρ*_*t*3–t1_ = − 0.02; *ρ*_*t*3–t2_ = 0.05	1−; 1−; 1−
			Vigorous: *ρ*_*t*2–t1_ = − 0.17; *ρ*_*t*3–t1_ = 0.06; *ρ*_*t*3–t2_ = 0.24	1−; 1−; 1−
			Sedentary: *ρ*_*t*2–t1_ = 0.39; *ρ*_*t*3–t1_ = 0.10; *ρ*_*t*3–t2_ = − 0.41	
AWAS [40]	52	Accelerometer (ActiGraph GT1M; right waist; waking hours of 7 days [3 days]; 5 s; Freedson et al. [78])	Total: *ρ* = 0.36 [0.12–0.57]	1−
			Light: *ρ* = 0.29 [0.03–0.51]	1−
			Moderate: *ρ* = 0.03 [− 0.26 to 0.32]	1−
			Vigorous: *ρ* = 0.43 [0.19–0.63]	1−
			MVPA: *ρ* = 0.03 [− 0.25 to 0.30]	1−
			Sedentary: *ρ* = 0.50 [0.25–0.69]	
			Classification (active/inactive)^e^: *κ* = − 0.09 [− 0.31 to 0.12]	
GPAQ [54]	95 *n*_t2_ = 85	Accelerometer (ActiGraph GTX3; right hip; waking hours of 7 days [3 days]; 15 s; Freedson et al. [78])	MVPA_t1_: $\bar{d}$= − 14.8, LOA^f^ = − 172.0 to 142.4 (min/day)	1−
			MVPA_t2_: $\bar{d}$= − 15.8, LOA^f^ = − 103.9 to 72.4 (min/day)	1−
			MVPA_t1_: *β*_0_^g^ = − 33.84 [− 78.49 to − 15.08], *β*_1_^g^ = 2.36 [1.61–4.05]	1−
			MVPA_t2_: *β*_0_^g^ = − 69.92 [− 238.84 to − 18.97], *β*_1_^g^ = 5.55 [2.62–16.95]	1−
			Sedentary_t1_: $\bar{d}$ = 127.5, LOA = − 299.2 to 554.2 (min/day)	
			Sedentary_t2_: $\bar{d}$= 89.2, LOA = − 390.7 to 569.2 (min/day)	
			Sedentary_t1_: *β*_0_ = − 1255.45 [− 2355.30 to − 694.92], *β*_1_ = 3.46 [2.18–5.84]	
			Sedentary_t2_: *β*_0_ = − 255.08 [− 672.24 to − 13.25], *β*_1_ = 1.45 [0.81–2.45]	
			Classification_t1_ (active/inactive)^h^: *κ* = 0.11, SE = 0.10	
			Classification_t2_ (active/inactive)^h^: *κ* = − 0.02, SE = 0.11	
			*Responsiveness*	
			MVPA: $\bar{d}$= 2.2, LOA^f^ = − 200.6 to 205.1 (min/day)	1−
			MVPA: *β*_0_^g^ = 103.92 [39.75–233.90], *β*_1_^g^ = 7.83 [3.80–19.62]	1−
IPAQ [48]	30	Accelerometer (ActiGraph GT1M; hip; waking hours of 5–7 days [5 days]; 60 s; Freedson et al. [78])	Total: *ρ* = 0.15	2−
			Total: $\bar{d}$ = 105.76, LOA^f^ = − 412 to 624 (MET-min/day)	2−
			Light: *ρ* = 0.03	1−
			Light: $\bar{d}$ = 255.55, LOA^f^ = − 10 to 511 (MET-min/day)	1−
			Moderate: *ρ* = 0.09	1−
			Moderate: $\bar{d}$ = − 112.25, LOA^f^ = − 445 to 220 (MET-min/day)	1−
		Pedometer (Yamax Digiwalker SW-700; hip; waking hours of 5–7 days [5 days]; 0.35 g threshold for one step)	Total: *ρ* = 0.30	3−
IPAQ-SF [52]	64	Accelerometer (SenseWear Armband: SWA Mini and SWA Pro 3; upper arm [left or right]; 8 days [4 days]; 10 min, moderate: 3–6 METs)	Moderate: *ρ* = 0.08 Vigorous: *ρ* = 0.39 MVPA: *ρ* = 0.14 MVPA: $\bar{d}$ = − 84.72, LOA^f^ = − 315.48 to 146.04 (min/wk)	1− 1− 1− 1−
LTEQ [53]	30	Pedometer (Yamax Digiwalker SW-701; waist; waking hours of 3 days [3 days], 2 periods: gw 20 and 32)	Total LT exercise: *r*_t1_ = 0.24; *r*_t2_ = 0.00	3−; 3−
			Mild LT exercise: *r*_t1_ = 0.13; *r*_t2_ = 0.13	3−; 3−
			Moderate LT exercise: *r*_t1_ = 0.35; *r*_t2_ = 0.19	3−; 3−
			Strenuous LT exercise: *r*_t1_ = 0.00; *r*_t2_ = 0.04	3−; 3−
LTPAQ [39]	47 *n*_ped_ = 45	Logbook (N/A)	Total LTPA (frequency): *ρ* = 0.27 [− 0.01 to 0.52]	3−
			Total LTPA (duration): *ρ* = 0.47 [0.21–0.67]	3−
			Light LTPA (frequency): *ρ* = 0.10 [− 0.20 to 0.37]	3−
			Light LTPA (duration): *ρ* = 0.21 [− 0.09 to 0.47]	3−
			MV-LTPA (frequency): *ρ* = 0.54 [0.30–0.72]	3−
			MV-LTPA (duration): *ρ* = 0.52 [0.28–0.71]	3−
		Pedometer (Omron HJ-113, Walking Style II; waking hours of 7 days [N/A], leisure time only)	Total LTPA (frequency): *ρ* = 0.16 [− 0.14 to 0.43]	3−
			Total LTPA (duration): *ρ* = − 0.18 [− 0.45 to 0.12]	3−
PAPQ [47]	77	Accelerometer (PreMed AS ActiReg; chest and front of right thigh; waking hours of 7 days [N/A]; PAL [moderate] = 1.61–1.90)	Light: *ρ* = 0.20	1−
			Moderate: *ρ* = 0.15	1−
			Vigorous: *ρ* = 0.59	1+
			Vigorous: $\bar{d}$ = 0.2, LOA^f^ = − 231.4 to 231.8 (min/wk)	1−
			Standing activities: *ρ* = 0.36	
			Standing activities: $\bar{d}$ = − 11.4, LOA = − 304.7 to 281.9 (min/day)	
			Sitting/lying: *ρ* = 0.29	
			Sitting/lying: $\bar{d}$ = 3.0, LOA = − 309.6 to 315.5 (min/day)	
			Classification (active/inactive)^h^: 97.4%; *ρ* = 0.47	
			Classification (quartiles): 84.5%; *ρ* = 0.39	
PPAQ [46] Bilingual version (English, French)	61	Pedometer (NL Digiwalker SW-200; waist; waking hours of 7 days [N/A])	Total: *r* = 0.36	3−
			Light: *r* = 0.26	3−
			Moderate: *r* = 0.27	3−
			Vigorous: *r* = 0.11	3−
			Household/caregiving: *r* = 0.12	3−
			Occupational: *r* = 0.26	3−
			Sports/exercise: *r* = 0.22	3−
			Sedentary: *r* = 0.03	
PPAQ [55] Chinese version	125	Accelerometer (Kenz Lifecorder, front hip; 7 days [3 days]; 11 activity levels [moderate = 4–6, Kumahara et al. [91]])	Total: *ρ* = 0.35	2−
			Light: *ρ* = 0.33	1−
			Moderate: *ρ* = 0.19	1−
			Vigorous: *ρ* = 0.15	1−
PPAQ [44] English version	54 *n*_occup_ = 38	Accelerometer (ActiGraph CSA; right hip; waking hours of 7 days [N/A]; 60 s; Freedson et al. [78], Hendelman et al. [79], Swartz et al. [77])	Total: *ρ* = 0.27	2−
			Light: *ρ* = 0.03	2−
			Moderate: *ρ* = 0.38	2−
			Vigorous: *ρ* = 0.37	2−
			Household/caregiving: *ρ* = − 0.04	2−
			Occupational: *ρ* = 0.16	2−
			Sports/exercise: *ρ* = 0.48	1−
			Sedentary: *ρ* = − 0.10	
PPAQ [42] English version	28	Accelerometer (Actical omniaxial; right hip; waking hours of 7 days [4 days]; 60 s; Colley et al. [81])	Light: *r* = 0.28	1−
			Moderate: *r* = 0.04	1−
			Vigorous: *r* = 0.43	1−
			MVPA: *r* = 0.02	1−
			LT-MVPA: *r* = 0.57	3+
			Sedentary: *r* = − 0.28	
			Classification (active/inactive)^h^: 34.4%	
PPAQ [43] French version	48 *n*_occup_ = 19	Accelerometer (ActiGraph GT1M; right hip; 7 days [N/A]; Matthews [80], Freedson et al. [78], Hendelman et al. [79], Swartz et al. [77])	Total: *ρ* = 0.58	2+
			Light: *ρ* = 0.53	2+
			Moderate: *ρ* = 0.49	2+
			Vigorous: *ρ* = 0.39	2−
			Household/caregiving: *ρ* = 0.56	2+
			Occupational: *ρ* = 0.56	2+
			Sports/exercise: *ρ* = 0.40	1−
			Transportation: *ρ* = 0.38	2−
			Sedentary: *ρ* = − 0.19	
PPAQ [49] Japanese version	54	Accelerometer (ActiGraph CSA; right hip; waking hours of 14 days [10 days]; 60 s; Freedson et al. [78], Hendelman et al. [79], Swartz et al. [77])	*1st measures*	
			Total^d^: *ρ* = 0.02; 0.35; 0.36	3−; 3−; 3−
			Light^d^: *ρ* = 0.01; 0.28; 0.34	3−; 3−; 3−
			Moderate^d^: *ρ* = − 0.09; 0.38; 0.30	3−; 3−; 3−
			Vigorous^d^: *ρ* = − 0.25; − 0.21; − 0.23	3−; 3−; 3−
			Sedentary^d^: *ρ* = 0.20; − 0.30; − 0.13	
			*2nd measures*	
			Total^d^: *ρ* = − 0.05; 0.29; 0.24	3−; 3−; 3−
			Light^d^: *ρ* = − 0.01; 0.13; 0.13	3−; 3−; 3−
			Moderate^d^: *ρ* = − 0.11; 0.38; 0.23	3−; 3−; 3−
			Sedentary^d^: *ρ* = 0.01; − 0.27; − 0.21	
			*3rd measures*	
			Total^d^: *ρ* = 0.04; 0.13; 0.20	3−; 3−; 3−
			Light^d^: *ρ* = 0.09; 0.05; 0.12	3−; 3−; 3−
			Moderate^d^: *ρ* = − 0.03; 0.15; 0.12	3−; 3−; 3−
			Vigorous^d^: *ρ* = − 0.18; − 0.09; − 0.18	3−; 3−; 3−
			Sedentary^d^: *ρ* = 0.20; − 0.12; 0.03	
PPAQ [45] Turkish version	204 *n*_ped_ = 85	Questionnaire (IPAQ-LF; N/A)	Total: *r* = 0.67	3−
			Light: *r* = 0.35	3−
			Moderate: *r* = 0.38	3−
			Vigorous: *r* = 0.42	3−
			Household/caregiving: *r* = 0.52	3−
			Occupational: *r* = 0.34	3−
			Sports/exercise: *r* = 0.02	3−
			Sedentary: *r* = − 0.65	
		Pedometer (Geonaute Onstep 100; waist; waking hours of 7 days [N/A])	Total: *r* = 0.70	3+
PPAQ [51] Vietnamese version	59	Pedometer (Digiwalker SW-200; waist; waking hours of 14 days [10 days])	Total: *r* = 0.29	3−
Q1 of MoBa [41]	112	Accelerometer (PreMed AS ActiReg; chest and front of right thigh; waking hours of 4 days [4 days]; TEE, PAEE, PAL, VPA calculated using ActiCalc [Hustvedt et al. [90]])	Total: *r *= 0.32	1−
RPAQ [40]	53	Accelerometer (ActiGraph GT1M; right waist; waking hours of 7 days [3 days]; 5 s; Freedson et al. [78])	Total (duration): *ρ* = 0.53 [0.32–0.70]	1+
			Total (EE): *ρ* = 0.18	2−
			Light: *ρ* = 0.41 [0.14–0.62]	1−
			Moderate: *ρ* = 0.06 [− 0.23 to 0.34]	1−
			Vigorous: *ρ* = − 0.03 [− 0.32 to 0.26]	1−
			MVPA: *ρ* = 0.06 [− 0.23 to 0.34]	1−
			Sedentary: *ρ* = 0.30 [0.03–0.54]	
			Classification (active/inactive)^e^: *κ* = 0.11 [− 0.09 to 0.31]	

*AQuAA* Activity Questionnaire for Adolescents and Adults, *AWAS* Australian Women’s Activity Study, $\bar{d}$ change in the mean, *EE* energy expenditure, *GPAQ* Global Physical Activity Questionnaire, *gw* gestational week, *IPAQ* International Physical Activity Questionnaire (long-form), *IPAQ-SF* International Physical Activity Questionnaire (short-form), *κ* kappa coefficient, *LOA* limits of agreement, *LT* leisure time, *LTEQ* Leisure-Time Exercise Questionnaire, *LTPAQ* Leisure Time Physical Activity Questionnaire (modified from IPAQ), *METs* metabolic equivalent of tasks, *min* minutes, *MV* moderate-to-vigorous, *MVPA* moderate-to-vigorous physical activity, *N/A* not applicable, *n*_*occup*_ sample size for occupational physical activity, *n*_*ped*_ sample size for comparison against pedometer, *PA* physical activity, *PAEE* physical activity energy expenditure, *PAL* physical activity level, *PAPQ* Physical Activity and Pregnancy Questionnaire, *ρ* Spearman correlation coefficient, *PPAQ* Pregnancy Physical Activity Questionnaire, *Q1 of MoBa* Questionnaire of recreational exercise from Norwegian, r Pearson correlation coefficient, *RPAQ* Recent Physical Activity Questionnaire, *SE* standard error, *t1* (or t2, t3) (multiple) measurements, *t1–t2* (or t2–t3, t3–t1) difference between two measurements, *TEE* total energy expenditure, *VPA* vigorous physical activity, *wk* week

^a^Cut points used in each individual study (i.e., Colley et al. [81], Freedson et al. [78], Hendelman et al. [79], Hustvedt et al. [90], Kumahara et al. [91], Matthews [80], Swartz et al. [77])

^b^When an individual study reported both results using different cut points and average (or total) counts, we integrated coefficients with higher quality

^c^As described in Sect. 2.6, the quality of the individual study was evaluated per questionnaire and PA score using four levels (1: very good, 2: adequate, 3: doubtful, 4: inadequate). Additionally, the reported results were rated (i.e., sufficient [+], insufficient [–]) as described in Sect. 2.5.3

^d^Spearman correlation (*ρ*) using Freedson et al. [78]; Hendelman et al. [79]; Swartz et al. [77] cut points

^e^‘Active’ if achieving 30 min of MVPA per day

^f^LOA between questionnaire and other measures of the same construct (e.g., accelerometer, pedometer) indicate the agreement between the two methods (as an indicator of construct validity and responsiveness), and therefore, was rated with the same criteria as described in the methods (Sects. 2.5.2, 2.6)

^g^Results from Passing Bablok regression [89], which indicated both proportional (slope [*β*_1_] significantly different from 0) and systematic (intercept [*β*_0_] significantly different from 1) differences between GPAQ and accelerometer estimated PA (minutes per day), led to a negative rating

^h^‘Active’ if achieving 150 min of MVPA per week

Of the seven questionnaires developed for the non-pregnant population, the AQuAA [50], AWAS [40], GPAQ [54], IPAQ [48], IPAQ-SF [52], and LTEQ [53] showed insufficient construct validity because of low coefficients or large disagreements (e.g., wide LOA). The RPAQ [40] showed a sufficient correlation with PA estimates from the accelerometer for total active time (*r* ≥ 0.50) but not for total physical activity energy expenditure (PAEE) and other estimates of PA.

Of the four questionnaires developed for the pregnant population, the LTPAQ [39] showed insufficient construct validity. The ratings for the PAPQ [47] were insufficient for light and moderate PA but sufficient for VPA. However, the LOA indicated large disagreement between PAPQ and accelerometry in assessing VPA. The results of studies of the construct validity of (versions of) the PPAQ were predominantly insufficient, such as for the Vietnamese [51], Japanese [49], English [44, 46], Chinese [55], and bilingual [46] versions of the questionnaire. Likewise, the second study [42] of the English version revealed insufficient construct validity for all scores expect for LT-MVPA. The Turkish version of the PPAQ [45] showed sufficient validity for the assessment of total PA due to a high correlation with the pedometer but insufficient ratings for all other estimates. The French version of the PPAQ [43] received sufficient ratings for total, light, and moderate PA, household/caregiving and occupational but insufficient ratings for sports/exercise, vigorous, and transportational PA. Finally, Q1 of MoBa [41] showed insufficient construct validity. There was a low correlation (*r* < 0.50) between sum of weekly exercise estimated from the questionnaire and VPA estimated from the accelerometer.

#### Responsiveness

Only two studies examined responsiveness for two questionnaires (see Table 5). The AQuAA [50] showed insufficient responsiveness. Similarly, the GPAQ [54] showed insufficient responsiveness because of large disagreements (large LOA*)* between the questionnaire and accelerometer. Moreover, the GPAQ showed both systematic (difference in intercepts) and proportional differences (difference in slopes) regarding the change in MVPA between 14–18 and 29–33 weeks of gestation as indicated by Passing Bablok regression [54].

### Quality of Individual Studies

Regarding the assessment of reliability of each PA score, nine studies [39, 40, 43–45, 49, 51, 52, 55] of AWAS, IPAQ-SF, LTPAQ, PPAQ, and RPAQ were at the highest level of quality (level 1) and one study [53] of the LTEQ at level 3 because of use of Pearson correlations and an inadequate time interval between test and retest. Regarding construct validity, six studies [40, 41, 47, 50, 52, 54] of AQuAA, AWAS, GPAQ, IPAQ-SF, PAPQ, and Q1 of MoBa were at the highest level of quality (level 1), four studies [40, 43, 44, 55] of PPAQ and RPAQ at level 1 and 2, one study [42] of PPAQ at level 1 and 3, and six studies [39, 45, 46, 49, 51, 53] of LTEQ, LTPAQ, and PPAQ at level 3 (see Table 5). The quality of one study of the IPAQ was either of level 1, level 2, or level 3 depending on the evaluated PA score [48]. Different levels of quality were assigned due to comparisons with either objective (e.g., accelerometer, pedometer) or subjective (e.g., logbook, questionnaire) measures of PA or comparisons between different intensity levels. For example, a lower level of quality was assigned if light PA measured by the questionnaire was compared with MVPA measured by the accelerometer (e.g., Japanese version of the PPAQ) [49] or if PA measured by the questionnaire was compared with pedometer measured daily steps (e.g., LTEQ) [53]. Furthermore, the quality for the assessment of total PA was often of level 2 because total PAEE estimated from the questionnaires was compared against accelerometer estimated total counts. Responsiveness was evaluated in two studies [50, 54] for two questionnaires (AQuAA, GPAQ). The quality of these studies was rated as level 1.

Finally, almost none of the studies formulated a priori hypotheses about expected results for construct validity or responsiveness. Only two studies [50, 52] of the AQuAA and IPAQ-SF considered a minimum correlation of *r* = 0.5 as an adequate agreement between PA questionnaire and accelerometer.

### Quality of Evidence

Table 6 summarizes the overall results (i.e., sufficient/insufficient measurement properties) and quality of evidence (GRADE) for three PA scores; total PA, MVPA, and VPA (per questionnaire and measurement property). None of the questionnaires provided evidence for all the relevant measurement properties (i.e., reliability [parameters of reliability or measurement error], construct validity, responsiveness). Only for the AWAS, IPAQ-SF, LTEQ, LTPAQ, PPAQ (i.e., Chinese, English, French, Japanese, Turkish, Vietnamese versions), and RPAQ was both reliability and construct validity assessed. Because there was usually only one study per questionnaire version and PA score available (except PPAQ), inconsistency could not be evaluated for these studies. With reference to the eligibility criteria and the checklist for methodological quality, we identified no serious indirectness, and therefore, did not downgrade the quality of evidence for any of the PA scores due to this factor.

**Table 6:** GRADE evidence profile: measurement properties of PA questionnaires for the assessment of total PA, MVPA and VPA during pregnancy

Measurement property	Outcome per questionnaire	Results	No. of studies (*n*^a^)	GRADE^b^			
				Risk of bias	Imprecision	Indirectness	Quality of evidence
Reliability							
	AWAS						
	Total	−	1 (56)	Serious	None	None	Moderate
	MVPA	−	1 (56)	Serious	None	None	Moderate
	VPA	−	1 (56)	Serious	None	None	Moderate
	IPAQ-SF						
	MVPA	+	1 (88)	Serious	None	None	Moderate
	VPA	+	1 (88)	Serious	None	None	Moderate
	LTEQ						
	Total	−	1 (37)	Very serious	Serious	None	Very low
	VPA	+	1 (37)	Very serious	Serious	None	Very low
	PPAQ Chinese version						
	Total	+	1 (125)	Serious	None	None	Moderate
	VPA	−	1 (125)	Serious	None	None	Moderate
	PPAQ English version						
	Total	+	1 (54)	Serious	None	None	Moderate
	VPA	+	1 (54)	Serious	None	None	Moderate
	PPAQ French version						
	Total	+	1 (49)	Serious	None	None	Moderate
	VPA	+	1 (49)	Serious	None	None	Moderate
	PPAQ Japanese version						
	Total	+	1 (58)	Serious	None	None	Moderate
	PPAQ Turkish version						
	Total	+	1 (204)	Serious	None	None	Moderate
	VPA	+	1 (204)	Serious	None	None	Moderate
	PPAQ Vietnamese version						
	Total	+	1 (60)	Serious	None	None	Moderate
	VPA	+	1 (60)	Serious	None	None	Moderate
	RPAQ						
	Total (EE)	−	1 (57)	Serious	None	None	Moderate
	Total (time)	−	1 (57)	Serious	None	None	Moderate
	MVPA	−	1 (57)	Serious	None	None	Moderate
	VPA	−	1 (57)	Serious	None	None	Moderate
Measurement error							
	LTPAQ						
	Total	−	1 (49)	Serious	None	None	Moderate
	MVPA	−	1 (49)	Serious	None	None	Moderate
Construct validity							
	AQuAA						
	Total	−	1 (55)	Serious	Serious	None	Low
	VPA	−	1 (55)	Serious	Serious	None	Low
	AWAS						
	Total	−	1 (52)	Serious	Serious	None	Low
	MVPA	−	1 (52)	Serious	Serious	None	Low
	VPA	−	1 (52)	Serious	Serious	None	Low
	GPAQ						
	MVPA	−	1 (95)	Serious	Serious	None	Low
	IPAQ						
	Total	−	1 (30)	Serious	Very serious	None	Very low
	IPAQ-SF						
	MVPA	−	1 (64)	Serious	Serious	None	Low
	VPA	−	1 (64)	Serious	Serious	None	Low
	LTEQ						
	Total	−	1 (30)	Very serious	Very serious	None	Very low
	VPA	−	1 (30)	Very serious	Very serious	None	Very low
	LTPAQ						
	Total	−	1 (47)	Very serious	Serious	None	Very low
	MVPA	−	1 (47)	Very serious	Serious	None	Very low
	PAPQ						
	VPA	+^c^	1 (77)	Serious	Serious	None	Low
	PPAQ Bilingual (English, French)						
	Total	−	1 (61)	Very serious	Serious	None	Very low
	VPA	−	1 (61)	Very serious	Serious	None	Very low
	PPAQ Chinese version						
	Total	−	1 (125)	Serious	None	None	Moderate
	VPA	−	1 (125)	Serious	None	None	Moderate
	PPAQ English version						
	Total	−	1 (54)	Serious	Serious	None	Low
	MVPA	−	1 (28)	Serious	Very serious	None	Very Low
	LT-MVPA	+	1 (28)	Very serious	Very serious	None	Very low
	VPA	−	2 (82)	None^d^	Serious	None	Moderate^e^
	PPAQ French version						
	Total	+	1 (48)	Serious	Serious	None	Low
	VPA	−	1 (48)	Serious	Serious	None	Low
	PPAQ Japanese version						
	Total	−	1 (54)	Very serious	Serious	None	Very low
	VPA	−	1 (54)	Very serious	Serious	None	Very low
	PPAQ Turkish version						
	Total	+^f^	1 (204)	Very serious	None	None	Low
	VPA	−	1 (204)	Very serious	None	None	Low
	PPAQ Vietnamese version						
	Total	−	1 (59)	Very serious	Serious	None	Very low
	Q1 of MoBa						
	Total	−	1 (112)	Serious	Serious	None	Low
	RPAQ						
	Total (EE)	−	1 (53)	Serious	Serious	None	Low
	Total (duration)	+	1 (53)	Serious	Serious	None	Low
	MVPA	−	1 (53)	Serious	Serious	None	Low
	VPA	−	1 (53)	Serious	Serious	None	Low
Responsiveness							
	AQuAA						
	Total	−	1 (31)	Serious	Very serious	None	Very low
	VPA	−	1 (31)	Serious	Very serious	None	Very low
	GPAQ						
	MVPA	−	1 (85)	Serious	Serious	None	Low

*AQuAA* Activity Questionnaire for Adolescents and Adults, *AWAS* Australian Women’s Activity Study, *EE* energy expenditure, *GPAQ* Global Physical Activity Questionnaire, *GRADE* Grading of Recommendation, Assessment, Development, and Evaluation, *IPAQ* International Physical Activity Questionnaire (long-form), *IPAQ-SF* International Physical Activity Questionnaire (short-form), *LT* leisure time, *LTEQ* Leisure-Time Exercise Questionnaire, *LTPAQ* Leisure Time Physical Activity Questionnaire (modified from IPAQ), *MVPA* moderate-to-vigorous physical activity, *PA* physical activity, *PAPQ* Physical Activity and Pregnancy Questionnaire, *PPAQ* Pregnancy Physical Activity Questionnaire, *Q1 of MoBa* Questionnaire of recreational exercise from Norwegian, *RPAQ* Recent Physical Activity Questionnaire, *VPA* vigorous physical activity

Results are shown as sufficient (+) or insufficient (−) measurement properties depending on scores and rating obtained from Tables 4 and 5

^a^Total number of participants, including the largest sample size per outcome in a particular study

^b^Inconsistency was not included in the table since it can only be evaluated when there are more than one study per outcome available. In our case this was only possible for the English version of the PPAQ (see Sect. 3.5)

^c^(−) when considering LOA (see Table 5)

^d^There was one study of very good (level 1) and one study of adequate (level 2) quality

^e^There was no serious inconsistency and/or indirectness

^f^Validation against pedometer

Overall and irrespective of the reported results (i.e., sufficient/insufficient measurement properties), the quality of the body of evidence was limited and ranged from very low to moderate. There was no high-quality evidence indicating that any of the included questionnaires had sufficient measurement properties in assessing total PA, MVPA, or VPA. Only the Turkish and French versions of the PPAQ showed both sufficient reliability and construct validity when assessing total PA (but not MVPA and VPA), but these results were based on low-to-moderate quality evidence.

Although different language versions of questionnaires should be treated initially separately [26], one may consider pooling the results (i.e., body of evidence) of the different versions of the PPAQ. When doing so, there was high-quality evidence (no serious risk of bias, no serious imprecision, no serious inconsistency, no serious indirectness) that the PPAQ had sufficient reliability in assessing total PA and VPA. We did not consider downgrading the quality of evidence for VPA as most of the results were sufficient (four of five studies), except the Chinese version, which may have occurred because most women did not engage in these activities, as suggested by the authors [55].

The results for construct validity of the PPAQ were inconsistent for total PA (i.e., two studies showed sufficient and five studies insufficient results) and consistently insufficient for VPA (see Table 6). When pooling these results, the PPAQ showed insufficient validity in assessing total PA, which was based on low-quality evidence (serious risk of bias, serious inconsistency, no serious imprecision, no serious indirectness). Similarly, there was moderate-quality evidence that the PPAQ has insufficient validity in assessing VPA (serious risk of bias, no serious inconsistency, no serious imprecision, no serious indirectness). We could not pool the results for MVPA and other measurement properties such as measurement error and responsiveness of the PPAQ due to a lack of multiple studies.

## Discussion

In contrast to the considerable evidence concerning measurement properties of PA questionnaires in adults [11], youth [10], and elderly people [12], little information is available about the quality of PA questionnaires in pregnancy. This article provides an overview of the measurement properties of all self-administered questionnaires assessing PA in pregnancy. In contrast to other reviews [66], the quality of individual studies as well as the overall quality of evidence was evaluated.

The findings show that the quality of evidence of measurement properties for self-administered PA questionnaires assessing PA in pregnancy is currently low to moderate. Most PA questionnaires showed insufficient measurement properties. Only two studies assessed responsiveness for two questionnaires (AQuAA, GPAQ) and, thus, no questionnaire demonstrated sufficiency for all relevant measurement properties (i.e., content validity, reliability, construct validity, responsiveness). Of those questionnaires for which evidence for both reliability and construct validity was available, only few showed consistent results. Based on low-to-moderate quality evidence, only the Turkish and French versions of the PPAQ showed sufficient reliability and construct validity in assessing total PA. When considering all versions together, the PPAQ showed sufficient reliability in assessing total PA and VPA, based on high-quality evidence. However, based on low-to-moderate quality evidence, the questionnaire showed insufficient construct validity in assessing these PA scores. Furthermore, the pooled results of the PPAQ were consistently sufficient for reliability, but inconsistent for construct validity (i.e., sufficient or insufficient). Although there was limited high-quality evidence, we currently recommend the PPAQ, irrespective of language, to assess PA during pregnancy. The PPAQ showed sufficient content validity and was the only included questionnaire with versions showing both sufficient reliability and validity.

Construct validity was assessed for all (versions of) questionnaires and most of them were compared with objective measures of PA such as accelerometers or pedometers. However, the methodological quality of these individual studies varied substantially. No study used DLW, although this technique can safely be applied in pregnancy [67], but it does not represent maternal EE since the DLW will cross the placenta. For many PA scores, comparisons were made with a different level of intensity in accelerometer data, which led to a lower quality of the individual study. For example, time spent in light activities does not necessarily correlate with time spent in moderate or vigorous activities. Furthermore, sometimes (total) PA was compared with pedometer estimated daily steps. Because pedometers are not able to capture duration, frequency, and intensity of PA [68], the quality of these individual studies was considered as doubtful. Only few studies reported statistics such as LOA to assess absolute validity, rather than relative validity evaluated with Spearman or Pearson correlations. Reliability was assessed for six questionnaires (11 versions) and the methodological quality of these individual studies was usually high. Most studies used ICC or LOA and adequate time intervals between test and retest. Finally, only two studies of very good quality assessed responsiveness, the ability of a questionnaire to detect changes in PA over time. Especially in pregnancy, a period in which PA usually changes profoundly [2], a questionnaire with sufficient responsiveness is needed to capture these changes.

During pregnancy, a precise focus on content validity such as the choice of recall periods, activities or relevant settings of PA is needed. First, the intensity, type, and duration of PA can change with the ongoing pregnancy [2]. For example, light activities become more frequent, especially during the second and third trimesters. Activities can become more intense throughout pregnancy because of increased fatigue [2] and energy requirements [69]. For example, carrying loads can be experienced as more exhausting in late compared to early pregnancy, and walking up the stairs will objectively require more energy with increasing body weight. Furthermore, work-related PA might be more important in early pregnancy compared to the second and/or third trimester due to maternity leave. Similarly, household and caregiving activities become more important, especially when assessing PA in combination with parity. These pregnancy-related changes should be considered when assessing PA during pregnancy. Questionnaires with sufficient content validity (AQuAA, AWAS, GPAQ, IPAQ, IPAQ-SF, LTPAQ, PAPQ, PPAQ), based on our elementary criteria, may need to be further appraised with respect to these considerations.

In pregnancy EE needed for some activities increases, especially in the second and third trimesters [69, 70], and the intensity of activities may be different [2, 71]. Many PA questionnaires use compendium-based information about MET intensities of different activities [64], which are based on the adult non-pregnant population. Pregnancy-specific MET intensities are scarce and may only be available for light and moderate household PA [72]. Such intensities are applied in, for example, the PPAQ. The lack of pregnancy-specific MET intensities together with the application of intensities from the non-pregnant population can be a source of bias when assessing total PA or PAEE. This could be the reason that for the RPAQ, a low correlation was shown for total PAEE, but a high correlation for total active time. However, more studies would be needed to test this hypothesis.

The present findings also revealed heterogeneity in the study design and analysis. This could result in a serious bias (e.g., risk of bias, inconsistency) and hampers the comparability of findings across (included) studies and countries. For example, accelerometers have been widely used to assess construct validity in this review. Although these devices can provide accurate information about duration, frequency, and intensity of PA under free-living conditions [73], there are currently no standards for accelerometer data collection and processing [74–76], including during pregnancy. Consequently, we observed large heterogeneity in data collection and processing criteria (Table 5). In contrast to the placement of the accelerometer (most women wore the device on their waist or hip), the included studies differed considerably in epoch length (i.e., 5 s to 10 min), registration period (3–14 days), and the definition of a valid week (e.g., 3 of 4 days, 4 of 8 days, 10 of 14 days). Furthermore, not all studies reported processing criteria, including the definition of filters and sampling frequency, which were reported least often. Since different decision rules for accelerometer data could impact PA outcomes [76], the reporting of these would increase transparency, comparability between studies and countries, and allow assessment of potential risks of bias.

Most importantly, we observed large heterogeneity in applied cut points [77–81] used to classify the intensity of PA into light, moderate, and vigorous. These cut points were usually developed for non-pregnant populations. For example, cut points for moderate PA in this review varied substantially between 191 [79] and 1952 [78] counts per minute, which will affect estimates of both PA and construct validity [82]. The influence of using different cut points on construct validity was demonstrated by two studies included in this review [49, 50]. Because there are currently no validated cut points available for pregnant women, it is unclear which cut points provide the best comparison for assessing construct validity. Not only are pregnancy-specific cut points lacking, but little is known in general about the reliability and validity of accelerometers in pregnancy [83]. Changes in body girth, gait, and monitor tilt can affect the accuracy and the ability to detect certain movements [84].

All things considered, objective devices such as accelerometers and pedometers are likely to provide sufficient reliability, whilst construct validity may be limited due to technical shortcomings, non-wearing time, participant interference with the results, and application of (different) cut points [85]. Lower construct validity of comparison measures clearly limits the quality of evidence for the validity of PA questionnaires. This is one of the greatest challenges for reviews on measurement properties of PA questionnaires, such as for the present review. Because of these shortcomings, future (validation) studies should report their decision rules in detail and attempt to develop guidelines for the optimal use of accelerometer data in the target population (e.g., pregnancy). To this end, two recent reviews emphasized the importance of such standards, as well as critically scrutinizing the validity of accelerometers and attempting to provide age-specific practical considerations for choosing the most appropriate method [85, 86].

### Recommendations for Choosing a Questionnaire

The choice of the right questionnaire depends on the study purpose. According to this, different settings (e.g., work, recreation), dimensions of PA (e.g., PAEE, total PA), or recall periods (e.g., last week, typical week) might become more important. In addition to previous recommendations for the selection of PA questionnaires [17], we recommend the following criteria for use in pregnancy:

(i) When assessing total PA, the questionnaire should cover all relevant settings of PA (work, home, transport, recreation, sports), but should especially focus on household/caregiving.
(ii) The questionnaire should measure at least duration and frequency of PA and should include a large range of light and moderate activities. Lower intensity activities become more prevalent during pregnancy, especially in the second and third trimesters. This will ensure sufficient content validity as well as discrimination of pregnant women regarding the level (e.g., time) engaged in these activities. For example, during the development of the PPAQ, light activities such as slowly walking at work while carrying light/moderate loads and childcare were one of the most discriminatory activities [44]. In general, identifying relevant activities for the target population should precede the selection of questions used.
(iii) The recall period of the questionnaire should be the last week (or last seven days), a typical week in a specific trimester, or the current trimester but should not expand over more than one trimester as PA during pregnancy varies [2].
(iv) Because pregnancy-specific MET intensities for different activities are lacking and energy cost changes during pregnancy, we further recommend using total time when assessing total PA instead of assigning activities different MET intensities from the non-pregnant population.

In general, we recommend using a questionnaire that has been evaluated in the target population and provides (consistent) results with sufficient content validity, reliability, construct validity, and responsiveness, based on high-quality evidence. If a questionnaire does not provide sufficient content validity, evaluation of further measurement properties is irrelevant. In our opinion, (versions of) the PPAQ may currently be the best choice to assess self-reported PA during pregnancy. However, some language versions of the PPAQ showed insufficient measurement properties, and, in fact, sufficient measurement properties for one language does not guarantee the same quality for other language versions and target populations. We carefully recommend not using AWAS, LTEQ, LTPAQ, RPAQ, and Q1 of MoBa (at least for some PA scores) because of insufficient content validity and/or both insufficient reliability and validity. However, our findings concerning the measurement properties of all included questionnaires were based on very low-to-moderate quality evidence.

### Limitations and Strengths of this Review

Whenever a study presented multiple PA scores for construct validity and responsiveness, we tried to integrate all of them into our tables. However, if an individual study used both different cut points and average counts, we integrated coefficients with higher quality (Table 1), usually average counts. Furthermore, we did not apply any restrictions concerning certain pregnancy characteristics such as parity or pregnancy body mass index (BMI). For example, study populations in this review consisted of both normal-weight and overweight/obese pregnant women. Whether this heterogeneity influenced the results is unclear and difficult to assess because of the low number of studies. However, in our review, this may have been a problem for only inter- and not intra-questionnaire comparisons.

Another problem was the observed heterogeneity in data collection and processing criteria of objective measures such as accelerometers and pedometers. Unfortunately, these criteria likely impact both PA and validation outcomes. We were unable to define particular criteria and comparison measures as a preferable ‘gold standard’. Although we tried to incorporate the use of accelerometer data and the similarity between constructs into our quality assessment, we did not evaluate the application of different decision rules such as registration period, epoch length, filter, valid wear time, and cut points. In theory, VPA estimated from the questionnaire should be compared with VPA measured by accelerometry but the use of different cut points influences this association. These limitations are of major concern for this systematic review. Since the results of the validity of a questionnaire strongly depend on the validity of the comparison measure, we recommend that all readers bear in mind the importance of standards when using objective measures of PA during pregnancy and interpret the presented results carefully.

Lastly, we tried to use state-of-the-art methodology for our quality and result rating. The assessment was based on our experience, a series of previous published systematic reviews [10–12], a standardized quality checklist for PA questionnaires [17] as well as the COSMIN [23, 26] and GRADE [25] guidelines. Researchers in the field are invited to discuss these findings in the light of their own expertise, possibly assigning different criteria (e.g., MIC of PA during pregnancy), levels of quality, and result ratings.

### Recommendations for Further Research

We recommend further studies assessing the quality of those questionnaires that provide sufficient content validity but limited high-quality evidence of sufficient measurement properties. Furthermore, future studies should include responsiveness in their assessment. In this review, most questionnaires were in the English language but a questionnaire should always be evaluated in the target population and language. We observed large heterogeneity in data collection and processing criteria. We strongly recommend that future studies be designed to develop standards for accelerometer use and analysis, in particular during pregnancy. Although only little is known about the validity of accelerometers in our target population, we currently recommend the use of omniaxial devices that capture all directions of movements and the use of total (or averaged) counts, which are independent from any cut points. Finally, since lower validity of (objective) comparison measures hinders the accurate estimation of the validity of a PA questionnaire, we strongly recommend research on the validity of accelerometers during pregnancy before evaluating measurement properties of PA questionnaires.

## Conclusions

Evidence concerning the measurement properties of self-administered PA questionnaires in pregnancy is at the moment limited and mostly of lower quality (i.e., very low to moderate). No questionnaire showed sufficient content validity, construct validity, reliability, and responsiveness. Some versions of the PPAQ showed sufficient measurement properties, based on low-to-moderate quality evidence. Overall (i.e., when pooling the results of all versions), the PPAQ showed sufficient reliability in assessing total PA and VPA, based on high-quality evidence. However, based on low-to-moderate quality evidence, the questionnaire revealed insufficient construct validity in assessing these PA scores. Only after the development of guidelines for the most appropriate use of accelerometer data during pregnancy will we be able to provide recommendations for PA questionnaires based on high-quality evidence.

## Electronic supplementary material

Below is the link to the electronic supplementary material.

Supplementary material 1 (DOCX 16 kb)
