# Supplementary material for: Physical Activity Questionnaires for Pregnancy: A Systematic Review of Measurement Properties
Source: Sports Med. 2018 Aug 9;48(10):2317–46. doi: 10.1007/s40279-018-0961-x (PMC6132497; doi:10.1007/s40279-018-0961-x)
Supplement: Supplementary file 1 — Supplementary material 1 (DOCX 16 kb) [file 40279_2018_961_MOESM1_ESM.docx]

Electronic Supplementary Material Appendix S1

Complete search strategy for PubMed

**Article title:** Physical Activity Questionnaires for Pregnancy: A Systematic Review of Measurement Properties

**Journal:** Sports Medicine

**Authors:** Matteo C. Sattler^*^, Johannes Jaunig, Estelle D. Watson, Mireille N.M. van Poppel, Lidwine B. Mokkink, Caroline B. Terwee and Pavel Dietz

*Correspondence: matteo.sattler@uni-graz.at

Institute of Sport Science, University of Graz, Graz, Mozartgasse 14, Austria

("motor activity"[Mesh:NoExp] OR Exercise[Mesh] OR Sports[Mesh] OR "Physical Exertion"[Mesh] OR "Early Ambulation"[Mesh] OR "Exercise Therapy"[Mesh] OR Motion[Mesh] OR "Movement Technique"[tiab] OR Motor Activit*[tiab] OR Physical Activit*[tiab] OR Locomotor Activit*[tiab] OR Exercis*[tiab] OR Training[tiab] OR Physical Condition*[tiab] OR “Physical Fitness”[tiab] OR “Physical Endurance”[tiab] OR “Movement Therapy”[tiab] OR “Fitness Training”[tiab] OR “Physical Training”[tiab] OR Plyometric[tiab] OR Weight-Lifting[tiab] OR Weight-Bearing[tiab] OR Running[tiab] OR Jogging[tiab] OR Walk*[tiab] OR Cycle[tiab] OR Cycling[tiab] OR Bicycl*[tiab] OR Rowing[tiab] OR Swim*[tiab] OR Ambulati*[tiab])

AND

(instrumentation[sh] OR methods[sh] OR Validation Studies[pt] OR Comparative Study[pt] OR "psychometrics"[MeSH] OR psychometr*[tiab] OR clinimetr*[tw] OR clinometr*[tw] OR "outcome assessment (health care)"[MeSH] OR outcome assessment[tiab] OR outcome measure*[tw] OR "observer variation"[MeSH] OR observer variation[tiab] OR "Health Status Indicators"[Mesh] OR "reproducibility of results"[MeSH] OR reproducib*[tiab] OR "discriminant analysis"[MeSH] OR reliab*[tiab] OR unreliab*[tiab] OR valid*[tiab] OR coefficient[tiab] OR homogeneity[tiab] OR homogeneous[tiab] OR "internal consistency"[tiab] OR (cronbach*[tiab] AND (alpha[tiab] OR alphas[tiab])) OR (item[tiab] AND (correlation*[tiab] OR selection*[tiab] OR reduction*[tiab])) OR agreement[tiab] OR precision[tiab] OR imprecision[tiab] OR "precise values"[tiab] OR test-retest[tiab] OR (test[tiab] AND retest[tiab]) OR (reliab*[tiab] AND (test[tiab] OR retest[tiab])) OR stability[tiab] OR interrater[tiab] OR inter-rater[tiab] OR intrarater[tiab] OR intra-rater[tiab] OR intertester[tiab] OR inter-tester[tiab] OR intratester[tiab] OR intra-tester[tiab] OR interobserver[tiab] OR inter-observer[tiab] OR intraobserver[tiab] OR intra-observer[tiab] OR intertechnician[tiab] OR inter-technician[tiab] OR intratechnician[tiab] OR intra-technician[tiab] OR interexaminer[tiab] OR inter-examiner[tiab] OR intraexaminer[tiab] OR intra-examiner[tiab] OR interassay[tiab] OR inter-assay[tiab] OR intraassay[tiab] OR intra-assay[tiab] OR interindividual[tiab] OR inter-individual[tiab] OR intraindividual[tiab] OR intra-individual[tiab] OR interparticipant[tiab] OR inter-participant[tiab] OR intraparticipant[tiab] OR intra-participant[tiab] OR kappa[tiab] OR kappa's[tiab] OR kappas[tiab] OR repeatab*[tiab] OR ((replicab*[tiab] OR repeated[tiab]) AND (measure[tiab] OR measures[tiab] OR findings[tiab] OR result[tiab] OR results[tiab] OR test[tiab] OR tests[tiab])) OR generaliza*[tiab] OR generalisa*[tiab] OR concordance[tiab] OR (intraclass[tiab] AND correlation*[tiab]) OR discriminative[tiab] OR "known group"[tiab] OR factor analysis[tiab] OR factor analyses[tiab] OR dimension*[tiab] OR subscale*[tiab] OR (multitrait[tiab] AND scaling[tiab] AND (analysis[tiab] OR analyses[tiab])) OR item discriminant[tiab] OR interscale correlation*[tiab] OR error[tiab] OR errors[tiab] OR "individual variability"[tiab] OR (variability[tiab] AND (analysis[tiab] OR values[tiab])) OR (uncertainty[tiab] AND (measurement[tiab] OR measuring[tiab])) OR "standard error of measurement"[tiab] OR sensitiv*[tiab] OR responsive*[tiab] OR ((minimal[tiab] OR minimally[tiab] OR clinical[tiab] OR clinically[tiab]) AND (important[tiab] OR significant[tiab] OR detectable[tiab]) AND (change[tiab] OR difference[tiab])) OR (small*[tiab] AND (real[tiab] OR detectable[tiab]) AND (change[tiab] OR difference[tiab])) OR meaningful change[tiab] OR "ceiling effect"[tiab] OR "floor effect"[tiab] OR "Item response model"[tiab] OR IRT[tiab] OR Rasch[tiab] OR "Differential item functioning"[tiab] OR DIF[tiab] OR "computer adaptive testing"[tiab] OR "item bank"[tiab] OR "cross-cultural equivalence"[tiab])

AND

((self[tiab] OR child[tiab] OR parent[tiab] OR proxy[tiab]) AND ((report[tiab] OR reported[tiab] OR reporting[tiab]) OR (rated[tiab] OR rating[tiab] OR ratings[tiab]) OR (assessed[tiab] OR assessment[tiab] OR assessments[tiab]))) AND (index[tiab] OR indices[tiab] OR instrument[tiab] OR instruments[tiab] OR measure[tiab] OR measures[tiab] OR questionnaire[tiab] OR questionnaires[tiab] OR profile[tiab] OR profiles[tiab] OR scale[tiab] OR scales[tiab] OR score[tiab] OR scores[tiab] OR status[tiab] OR survey[tiab] OR surveys[tiab])

AND

(Pregnancy[Mesh] OR Gravidity[Mesh] OR “Pregnant Women”[Mesh] OR Pregnan*[tiab] OR Gravid*[tiab] OR Gestation*[tiab] OR Childbearing[tiab] OR Matern*[tiab] OR Perinat* [tiab] OR Antenat*[tiab] OR Parit*[tiab])

NOT

(addresses[PT] OR biography[PT] OR “case reports”[PT] OR comment[PT] OR directory[PT] OR editorial[PT] OR festschrift[PT] OR interview[PT] OR lectures[PT] OR “legal cases”[PT] OR legislation[PT] OR letter[PT] OR news[PT] OR “newspaper article”[PT] OR “patient education handout”[PT] OR “popular works”[PT] OR congresses[PT] OR “consensus development conference”[PT] OR “consensus development conference”, nih[PT] OR “practice guideline”[PT]) OR (animals[MeSH Terms] NOT humans[MeSH Terms])
